# Experiences with Innovation Fund healthcare models in primary care: a qualitative study amongst German general practitioners

**DOI:** 10.1007/s10354-022-00935-0

**Published:** 2022-05-03

**Authors:** Julian Wangler, Michael Jansky

**Affiliations:** Centre for General Medicine and Geriatrics, University Medical Center of the Johannes Gutenberg University Mainz, Am Pulverturm 13, 55131 Mainz, Germany

**Keywords:** Innovation Fund, Health services research, General practitioner, Primary care, Recruitment, Innovationsfonds, Versorgungsforschung, Hausarzt, Primärversorgung, Rekrutierung

## Abstract

The Innovation Fund was set up in 2015 with the aim of improving medical care in the German statutory health insurance system. Primary care needs to be involved in testing interventions and new forms of care for effectiveness and inclusion in standard care. There has so far been hardly any research on how far Innovation Fund models accommodate the primary care setting, or on the experience general practitioners have had with these models. Between September 2021 and January 2022, 36 semi-standardized individual interviews were performed with general practitioners who had already participated in Innovation Fund projects. Eleven regional physician networks in Rhineland-Palatinate, Hesse, North Rhine-Westphalia, and Schleswig-Holstein were involved in the recruitment process. Most of the interviewees associated the Innovation Fund with potential and opportunity including intensification of application-oriented healthcare research, independent financing, and general healthcare involvement. Even so, many general practitioners were unsure as to how far primary care in particular would stand to benefit from the Innovation Fund in the long term. A mostly positive balance was drawn from participation in care models—benefit of intervention as well as cost–benefit ratio. However, some also reported hurdles and stress factors such as documentation requirements and disruption in everyday office routine. Innovation Fund projects will need to be suitable for general practitioners especially regarding medical decision-making leeway, limits to documentation requirements, preserving established office routine, greater involvement in research planning, and improvements to the primary care setting to encourage willingness to participate in Innovation Fund projects amongst general practitioners.

**Supplementary Information:**

The online version of this article (10.1007/s10354-022-00935-0) contains supplementary material, which is available to authorized users.

## Abstract (de)

Im Jahr 2015 wurde in Deutschland zwecks Verbesserung der medizinischen Versorgung innerhalb der gesetzlichen Krankenversicherung der Innovationsfonds etabliert. Zur Erprobung und Wirksamkeitskontrolle von Interventionen und neuen Versorgungsformen kommt es maßgeblich auf die Involvierung der hausärztlichen Versorgung an. Bislang fehlt es an Studien, die der Frage nachgehen, inwiefern Innovationsfondsstudien dem hausärztlichen Setting gerecht werden und welche Erfahrungen Hausärzt*innen mit solchen Projekten gemacht haben. Zwischen September 2021 und Januar 2022 wurden 36 halbstandardisierte Einzelinterviews mit Hausärzt*innen geführt, die bereits Innovationsfondserfahrung sammeln konnten. Die Interviewten sehen in der Einrichtung des Innovationsfonds Chancen und Entwicklungsperspektiven, gerade im Hinblick auf eine Intensivierung anwendungsnaher Versorgungsforschung, unabhängige Finanzierung sowie Einbeziehung der Breitenversorgung. Nichtsdestotrotz artikulieren viele Hausärzt*innen Unsicherheit, ob der Innovationsfonds dauerhaft zu positiven Effekten für die hausärztliche Versorgung wird führen können. Die Erfahrungen, die infolge der Teilnahme an den Versorgungsmodellen gesammelt wurden, werden als mehrheitlich positiv beschrieben (v. a. Nutzen der Intervention, Aufwand-Nutzen-Verhältnis). Zugleich wird auch über Belastungsfaktoren und Probleme berichtet, etwa Dokumentationspflichten und Beeinträchtigung von Praxisabläufen. Um mehr Hausärzt*innen für eine Teilnahme an Innovationsfondsprojekten zu gewinnen, kommt es darauf an, die Hausarztkonformität solcher Studien zu erhöhen, v. a. mit Blick auf ärztliche Entscheidungsspielräume, die Limitierung von Dokumentationspflichten, die Gewährleistung von Praxisroutinen, eine stärkere Involvierung in die Forschungsplanung sowie eine Aufwertung des hausärztlichen Settings.

## Introduction

Optimizing medical care in the long term will require new forms of care to be developed and tested, and theoretical and applied healthcare research to be reinforced [1]. Complex clinical research also requires steady independent funding. The German Federal Joint Committee (G-BA) adopted the Innovation Fund 2015 in order to meet these requirements [2, 3]. As a health policy instrument, the aim of the fund is to promote evidence-based development in pay-as-you-go healthcare by developing new healthcare concepts [2, 4, 5]. An annual funding volume of € 200 million has been secured for the project in the current funding phase, with funding provided by the statutory health insurance companies and health fund. This places the Innovation Fund inside a small group of similar initiatives with a comparable purpose worldwide [6].

Projects receiving this form of funding have so far included healthcare models in rural areas, special patient groups, drug therapy, delegation models, and reinforcement of cross-sector healthcare services. One important requirement in the Innovation Fund was that funded projects would need to be suitable for permanent implementation regarding the healthcare form developed [2, 7]. To do this, they would need to be scientifically evaluated to prove their effectiveness as an intervention. A successful project would then stand a chance of being adopted in standard healthcare or selective contracts [4].

Primary care is an important focal point for interventions and healthcare concepts benefiting from the Innovation Fund. Cooperation with primary care often plays an important role considering that new care models need to involve a sufficiently large cohort of patients to establish their efficacy to a sufficient degree, especially in a low-prevalence context [8, 9]. The Innovation Fund theoretically provides an opportunity for general practitioners (GPs) to benefit especially from new forms of healthcare [10]. However, this potential is dampened by the challenge of recruiting enough general practitioners for corresponding research projects that often require a complex cluster-randomized design (randomized clinical trials [RCTs]). This begs the question as to how far Innovation Fund models are compatible with a primary care setting and can be implemented here [11].

Elucidating the perspective of general practitioners in private practice will play a major role in addressing attitudes, empirical values, and potential issues in involving general practitioners in Innovation Fund projects. There have so far not been any studies on the attitudes of general practitioners towards the Innovation Fund and the experiences they have had with project participation.

## Materials and methods

### Study design and setting

The present study pursued an explorative approach implemented with the help of qualitative, semi-standardized interviews in addressing the research purpose outlined towards gaining a general impression of the opinions and experiences of physicians in primary care. The aim of the study was to recruit general practitioners with Innovation Fund experience.

### Interview guidelines

The guidelines were developed taking into account a literature review (including Lech et al. [10] and Heytens et al. [4]) and the authors’ own research and recruitment experience regarding the Innovation Fund [12, 13]. These guidelines consist of four key areas:

- Attitudes towards Innovation Fund projects and their benefits
- Willingness to participate and corresponding prerequisites
- Experiences from taking part in specific projects
- Perceived optimization potential

### Recruitment, participants, and sampling

The authors launched an appeal (letter by post) via 11 regional doctor networks in Rhineland-Palatinate, Hesse, North Rhine-Westphalia, and Schleswig-Holstein on a study aiming to gather opinions and experiences from general practitioners who had already participated in Innovation Fund projects.

A total of 36 general practitioners responded, and the interviews were ultimately conducted with these physicians (without incentives). Details of the sample are provided in Table 1.

**Table 1:** Survey emphases and sociodemographic factors in the survey sample (*N* = 36)

Type of office	18 joint offices, 18 single offices
Office environment	8 in small towns or rural communities, 15 in medium-sized towns, 13 in cities
Status	28 offices owned by the GP, 8 GPs in employment
Age	Average 52 years old
Gender	23 male, 13 female

A similarly heterogeneous sample was obtained in comparison to specific Innovation Fund projects in which general practitioners were to be recruited [14].

All 36 interviews were conducted verbally and personally by the authors in turn between September 2021 and January 2022 (interview duration: 40–70 min). Respondents were sent an explanation of the topic and declaration of consent to sign before the interview, and additional information such as assurance of anonymization.

### Data analysis

After transcription, we evaluated the interviews on the basis of the qualitative content analysis according to Mayring [15] in a team using the MAXQDA (VERBI Software, Berlin, Germany) software. In preparation, the written consultations were summarized with the essential information to gain an overview of the fundamental material. The text was then extracted in individual sentences or paragraphs depending on importance and expressiveness, with units to be used in analysis previously determined (context, interview code, original text, paraphrasing, generalization). The most important core statements were isolated, abstracted, and summarized before forming categories. The categorical system (see Multimedia Appendix 1 in the Supplementary Material) created was structured closely to the guideline and repeatedly reviewed and modified as necessary during evaluation. Here, we focused on forming logical categories from the various opinions and experiences. We used the COREQ methodology in the reporting statement. In the results section, terms such as “(I-8 m)” after quotes reflect the anonymization process.

## Results

### Background to the Innovation Fund commitment

Almost all interviewees reported that they had already been familiar with the German institution concerned before taking part in its Innovation Fund project. However, 10 respondents stated that they had not known that general practitioners were explicitly able to participate in model studies on the Innovation Fund before being recruited.

While 16 respondents in the sample had been asked about possible participation in the Innovation Fund once for the purpose of recruitment, 12 others had been asked twice. Eight respondents named more than two recruitment attempts for different projects. Regardless of recruitment attempts, 25 stated that they had previously participated in a project or were currently involved in one, and 11 respondents named two or more projects. Most of the involvement involved certain public healthcare insurance companies.

The projects were focused on optimizing specific patient healthcare services; drug therapy and drug therapy safety; extension to regional, multiprofessional, and cross-sectoral care structures; and promotion of evidence-based medicine and adherence to guidelines. A large share of projects related to telemedicine also emerged.

### Attitudes towards Innovation Fund projects

Most of the interviewees gave the establishment of the Innovation Fund a positive rating. Around every third interviewee perceived great benefit for patient care, another third saw a moderate benefit.

Of course, you have to be careful not to overburden this idea with expectations in too short a time. Even so, I still think it gives us an opportunity to benefit from targeted and sustainable improvements in taking care of our patients. (I-8 m)

Many of those interviewed associated the Innovation Fund with opportunities for the healthcare system, especially regarding independent financing and identifying or closing gaps in healthcare. Most also welcomed the opportunity for general practitioners to become more involved in scientific research.

Complex clinical research—Germany has long since been a bit of a developing country in getting general practitioners on board. This is where the vast majority of patients receive healthcare in everyday life. […] So, it’s definitely a step in the right direction. (I-17 f)

I think it’s especially important that these large sums of money for research are now coming from a neutral place. […] We need to get away from agenda-based science. (I-19 m)

Despite the perceived potential in the Innovation Fund, some of the interviewees raised concerns about negative effects and the possibility of bypassing important issues, such as in addressing requirements of primary care. Almost every second respondent in the sample expressed doubts as to whether Innovation Fund projects would be able to contribute to an improvement in primary care in the long term.

The whole thing could also have a negative side. […] For example, I see a risk that these studies might ultimately bypass the reality of general practitioners too much and be of little use to us […], or even a burden in the worst case. (I-11 m)

We’ll have to see how sustainable these interventions are in overall healthcare. (I-17 f)

Some of the interviewees were apprehensive in that general practitioners could “be used more as a stopgap” (I-8 m) in the Innovation Fund context by providing their patients and trying out interventions without sufficient involvement opportunity themselves.

We’ve already seen that happen. General practitioners were recruited, but more as a means to an end. (I-14 m)

Some respondents were skeptical about the Innovation Fund “as a politically devised institution” that “doesn’t necessarily support projects that the healthcare system needs” (I-11 m). The concern was raised of “over-engineering and bloating the healthcare system […] at the expense of the primary care budget” (I-24 m) from personal experience in projects establishing new healthcare protagonists such as specialized case managers. It was also unclear whether Innovation Fund models would “actually find their way into standard healthcare” (I-28 f).

### Willingness to participate and prerequisites

The main reasons interviewees gave for participation included curiosity and involvement in scientific research and a desire to contribute to improvements in patient healthcare and quality of life. However, some interviewees make it clear that they initially weighed up the feasibility of taking part in a large scientific study considering their heavy workload.

The respondents named various requirements for participating in (future) Innovation Fund studies. Apart from likely diagnostic or therapeutic benefit, they mainly focused on issues regarding the (limited) additional burden (such as preparation and follow-up, documentation, patient registration), appropriate remuneration, and structural improvement to the primary care setting. Interviewees also pointed out the importance of projects not causing any major changes to office routine and the responsibilities involved as well as optimized cooperation between the healthcare levels.

Committing yourself to studies like this isn’t trivial. They should see how they can accommodate general practitioners here. I think there’s still too little of that. (I-12 f)

### Experiences from taking part in specific projects

Some general practitioners reported that they themselves had only become aware of their Innovation Fund project and signed up for it in response to reading an announcement, or similar. Others mentioned recruitment from project staff or partners, such as the respective physicians’ network or public health insurance company.

More than half the interviewees reported that they had needed to train office staff as a result of participating in the project. Overall, two-thirds of the doctors stated that their participation in the project had led to temporary moderate to severe limitations or inconveniences in office routine.

That was an issue. The office staff had to undergo a huge amount of preparation, the short-term training requirement was heavy […] we were not informed about the type and extent of training from the start, and the training was scheduled at too short notice. This made normal office routine more difficult. (I-32 f)

One of the common problems mentioned is that general practitioners were required to carry out a diagnostic procedure that was based on guidelines or recommendations for action from other disciplines that did not conform to primary care. Especially in projects that worked with the intervention of interprofessional actors (e.g., case managers), it was difficult to reconcile the interaction with these actors with regular practice. There were often delays and obstacles in normal patient care.

Overall, interviewees drew a positive conclusion regarding the benefits of the intervention tested. More than two-thirds saw a (very) strong benefit to healthcare or therapy for patients involved. A similar share of the interviewees saw the benefits of project participation outweighing the effort. Projects covering healthcare in economically underdeveloped areas, drug therapy/safety, delegation, and substitution as well as cross-sector healthcare were assessed positively for added value.

The physicians were satisfied with the (intervention) results they saw, improved cooperation with other healthcare services, and development of diagnostic and therapeutic skills. Negative points included documentation requirements and heavy bureaucratic burden, disruption in office routine and, from the point of view of some doctors, insufficient involvement in research processes and project-related decisions. A few reported pressure from the project management to “recruit an unrealistically large patient cohort” (I-8 m).

The hurdles and additional burdens shouldn’t be underestimated. I can understand why not all doctors can take part. (I-31 m)

Four respondents who had already taken part in Innovation Fund projects reported that they had already left the project due to overload. The main reasons were excessive additional burden, (documentation) effort, and interference with office routine.

This project just got out of hand. They constantly raised the requirements for me as a doctor without consulting me about it. It became too much of a burden at a certain point. (I-36 f)

Even so, most of the respondents stated that they would generally consider participating in other Innovation Fund projects in the future provided the project promised worthwhile benefits for primary care from their point of view.

### Potential for optimization

Speaking from their experience in projects, the interviewees named a number of improvements they would like to see. These involved a limit to documentation requirements, more organizational clarity with regard to project coordination, opportunity for more flexibility in medical decision-making (such as inpatient appointments and therapy-related decisions), less disruption in office routine, and reinforcement and improved structure in communication and collaboration with other healthcare providers. Finally, the participating physicians stated that they would appreciate (more) cost-based remuneration. The responses also demonstrate that the position of general practitioners should be reinforced further in various Innovation Fund project stages.

General practitioners simply need to be more involved than before in designing and developing new studies and healthcare models. (I-29 f)

## Discussion

### Main findings and interpretation

The study results show that most of the general practitioners interviewed gave a positive rating to the experience they had gained with the Innovation Fund and saw opportunities in the concept. On the other hand, we sometimes perceived a critical and distanced attitude concerning long-term goal orientation and the actual benefit for primary care from corresponding studies. On balance, project participation generally fetched positive reactions (therapy quality, cost–benefit ratio). Even so, many of the respondents reported stress factors involving documentation requirements and administrative effort, changes in office routine, and remuneration that was not always considered commensurate.

The results from the survey may be seen as confirmation that general practitioners are now more willing to participate in empirical, evidence-oriented studies to optimize healthcare [16]. Even so, various studies have reported a significant number of general practitioners not being available for research projects of this type [8–10]. This has resulted in a shortage of general practitioner recruitment in complex studies as especially project experience involving the Innovation Fund demonstrates, with studies often failing to reach the target number of physicians and patients originally intended [17, 18].

The reasons for the hurdles and challenges to recruiting general practitioners for Innovation Fund and other complex clinical studies have hardly been investigated up to now, but there are indications that these projects do not easily combine with everyday primary care. Apart from a fundamental lack of time and resources being an issue [19], general practitioners have voiced concerns about logistical feasibility and uncontrolled increases in additional workload as well as occasional doubts as to the integrability or compatibility of interventions in everyday primary care and the incentive structure in clinical studies [19–26]. Problems that were widely addressed in the course of the interviews concerned, for example, the commitment of general practitioners to specialist guidelines that did not conform to general practitioners. In addition, interaction with interprofessional actors, who are used in some of the Innovation Fund projects to improve cross-sectoral care, caused interaction and organizational problems.

In one review, Fletcher et al. [27] identified barriers such as poor communication from study coordinators, difficulties amongst general practitioners in understanding research methods, concerns about possible harm to patients, and a sense of being overwhelmed by too many research requests without being seen as a fully legitimate research partner. Lech et al. [10] discussed requirements for a specific recruitment of general practitioners. It has been emphasized that this could be optimized in RCT studies with a greater concentration on regional, thematically specialized physician networks [28, 29].

The wishes and requirements expressed by respondents for participation in Innovation Fund studies agree with other studies, demonstrating possibilities towards recruiting more general practitioners in the future. General practitioners look for more freedom for individual decision-making flexibility and limits to administrative effort while keeping to established office routine from participating not only in research activities, but also in evidence-based structures and instruments [21]. GP compliance is therefore a crucial factor if the seamless integration of GPs into such research projects and thus their long-term satisfaction is to be guaranteed. An example of this is the involvement of multiprofessional actors such as case managers who interact with general practitioners in the Innovation Fund context and take on specific tasks. Here it must be planned in advance how contacts and contact rhythms can be designed in such a way that the everyday practice of general practitioners is not disturbed as far as possible and synergy effects can be developed [8, 9, 20]. Apparently, this is not the case for all projects. In addition, it is a reality that Innovation Fund studies are often projects initiated by specialists in which general practitioners are involved (e.g., for subprojects or to test a diagnostic intervention). Accordingly, general practitioners are often given a procedure that originates from the guidelines and evidence-based instruments of other disciplines and therefore causes problems in their own patient care or overwhelms general practitioners (e.g., when thinking of a specialist area such as the clarification of elevated liver values) [16, 30].

There is also a desire for an opportunity or more of an opportunity to help shape project activities. This tallies with the results from previous surveys in research primary care practice networks (see [19–21, 31, 32] for examples). All this indicates that clinical research projects have still not always been compatible with the primary care setting up to now [9, 18, 20, 33].

### Strengths and limitations

The present study is one of the first to deal with the issue of attitudes and experience of general practitioners towards Innovation Fund studies. However, the study cannot make any representative claims due to the limited number of cases and regional recruiting focus. The present work is unique to date, as it looks at Innovation Fund experiences of general practitioners from a higher-level perspective. A similarly heterogeneous sample was obtained in comparison to specific Innovation Fund projects in which general practitioners were to be recruited. For example, a recently published study conducted in the region of Berlin and Brandenburg described quite similar demographic characteristics of GPs [14].

Apart from that, all general practitioners recruited were members of a physicians’ network. For the above reasons, the present study cannot make any statements about general practitioners taking part in the Innovation Fund outside these networks or about the large number of general practitioners without previous project experience involving the Innovation Fund. Besides, there is another important reason for potential lack of generalizability of the present results: participants of the present study are GPs who participated in an Innovation Fund project. Thus, it is reasonable to believe that their opinions and attitudes on the Innovation Fund are somewhat more positive than those of GPs who decided not to participate.

The large number of general practitioners not or not yet available for Innovation Fund studies suggests that future studies will need to focus mainly on this group regarding their attitudes, expectations, and desires, while also developing measures to make Innovation Fund participation more attractive. This immediately begs the question as to which attitudes might keep general practitioners from participating in care models.

The present study has not looked into how far the projects the responding general practitioners took part in were implemented, comanaged, or coordinated by primary care institutes. These institutes have gathered a wealth of experience in research collaboration with general practitioners. Future studies should therefore focus on whether the study conditions for general practitioners could be more favorable in cooperation with primary care institutes.

The present study has also not assessed the extent to which settings other than clinical studies involving the Innovation Fund may be more suited to primary care regarding willingness to become involved in scientific research. Studies from primary care suggest that the research practice model may potentially achieve better recruitment and participation [9, 22, 25, 33–35]. In this respect, results from the present study may be compared with results from a survey to be suggested here documenting the experiences of general practitioners specifically in the research practice setting. This type of survey would be feasible on a larger scale in view of the recent emergence of larger research networks coordinated by primary care institutions.

## Conclusion

The interviews provide an insight into how general practitioners perceive the Innovation Fund from their existing project experience and what requirements studies would have to fulfil for general practitioners to participate. In particular, projects would need to be compatible with the primary care setting, especially in providing medical decision-making discretion, limits to documentation requirements, disruption in office routine, greater involvement in research planning, and longer-term improvement in primary care. Innovation Fund projects should be designed and communicated for their clear relevance to everyday primary care [9, 33–36].

### Supplementary Information

Appendix 1: Categorical system
