# Supplementary material for: Experiences with Innovation Fund healthcare models in primary care: a qualitative study amongst German general practitioners
Source: Wien Med Wochenschr. 2022 May 3;174(3-4):53–60. doi: 10.1007/s10354-022-00935-0 (PMC10896771; doi:10.1007/s10354-022-00935-0)
Supplement: Supplementary file 1 — Appendix 1: Categorical system [file 10354_2022_935_MOESM1_ESM.docx]

**Appendix 1: Categorical system:**

**1) Attitudes towards Innovation Fund projects and perception of their benefits:**

a) Principal benefit for the improvement of (regular) care

b) Neutral research funding vs. political influence

c) Application-oriented or sustainable optimization of incorrect or incomplete care

d) Primary care physician participation in clinical research

e) Addressing of primary care needs and the accuracy of interventions

f) Position of general practitioners in the context of Innovation Fund-driven research

g) Structural changes of healthcare due to Innovation Fund-driven interventions

**2) Willingness to participate and its requirements:**

a) Interest in or participation in patient-related and clinical research

b) Optimization of patient care and quality of life as well as diagnostic and/or therapeutic benefits

c) Effort and burden

d) Remuneration

e) (Structural) revaluation of primary care work

f) Changes in work processes, practice routines and responsibilities

g) Improving cross-sector and multi-professional care

h) Training or development of diagnostic or therapeutic skills

i) Optimization of structuring and efficiency of patient care

j) Better anticipation of care crises

**3) Experiences in participating in specific projects:**

a) Type of recruitment or enrollment

b) Training needs with regard to the practice staff

c) Changes or restrictions in practice operations due to project participation

d) Experiences and observations with regard to the benefit of the intervention

e) Effort-benefit ratio of project participation

f) Assessment of individual elements of project participation

g) Complete termination of project participation or premature termination

h) Willingness to participate in future Innovation Fund projects against the backdrop of experiences made

**4) Perceived optimization potential:**

a) Limiting administrative effort

b) Organizational structuring and project coordination

c) Enabling GP decision-making flexibility

d) Limitation of interventions in practice processes

e) Management of interdisciplinary communication

f) Effort-based remuneration

g) (More) involvement of general practitioners in project design and implementation
